# Comparison of the Cracking Behavior of Powder Metallurgy and Ingot Metallurgy Ti-5Al-5Mo-5V-3Cr Alloys during Hot Deformation

**DOI:** 10.3390/ma12030457

**Published:** 2019-02-01

**Authors:** Qinyang Zhao, Fei Yang, Rob Torrens, Leandro Bolzoni

**Affiliations:** Waikato Centre for Advanced Materials, School of Engineering, University of Waikato, Hamilton 3240, New Zealand; qz109@students.waikato.ac.nz (Q.Z.); rob.torrens@waikato.ac.nz (R.T.); leandro.bolzoni@waikato.ac.nz (L.B.)

**Keywords:** powder metallurgy, Ti-5553 alloy, hot deformation, cracking behavior

## Abstract

The hot workability of metallic materials is significantly dependent on its ability to form plastic without cracking and fracturing. In this work, the cracking behavior of powder metallurgy (PM) Ti-5Al-5Mo-5V-3Cr (Ti-5553) alloy, consolidated from powder mixture, at a deformation temperature range of 600 °C–850 °C and strain rate of 0.1 s^−1^–10 s^−1^, was investigated through isothermal compression tests. The cracking behavior of the as-cast ingot metallurgy (IM) Ti-5553 alloy, at a deformation temperature of 700 °C was also investigated for comparison. Results suggested that the PM Ti-5553 alloy had a better hot workability, with a larger cracking-free processing window, and a lower deformation resistance than the IM counterpart. 45° shear fracture occurred in the PM alloy, compressed at the condition of 600 °C/10 s^−1^, and edge cracking was observed at the 700 °C/10 s^−1^. 45° shear fracture was also significant in the IM alloy specimen tested at 700 °C/10 s^−1^, and all the other IM alloy specimens compressed at 700 °C displayed longitudinal cracking. Moreover, the microscopic cracking observation showed that ductile dimple cracking can be found in the IM alloy, but brittle cleavage fracture was dominant in the cracking surface of PM alloy with a relatively low cracking ductility.

## 1. Introduction

Titanium and its alloys are attractive materials in aerospace, chemical, marine, and civil applications, due to their excellent properties, including low density, high specific strength, good corrosion resistance, and great high-temperature properties [1,2,3,4,5,6]. Ti-5553 (Ti-5Al-5Mo-5V-3Cr) is a near beta high strength titanium alloy and was developed from the Russian VT-22 alloy, for the application of aerospace industry, particular in thick forged components (such as the Boeing-787 and Airbus-350 aircrafts’ landing gears and flap tracks), due to its ultra-high strength, good fatigue resistance, and deep hardenability [7,8,9,10,11].

However, widespread use of titanium alloy products is significantly limited by their high cost, as a result of its multi-step hot processing and further machining [12,13,14,15]. The powder metallurgy (PM) approach has been verified to be a cost-effective processing technique to produce titanium alloy products that meet the requirement of the industrial applications, with additional benefits, such as the manufacture of near-net-shape parts and optimization of the microstructure [13,16,17]. Furthermore, utilizing rapid consolidation processing methods, such as hot pressing and powder forging, instead of conventional vacuum sintering and hot isostatic pressing (HIP) processes, the titanium alloy products can be produced in a much shorter process and their costs can be reduced significantly [18,19].

In general, cracking and fracturing are important factors that influence the hot workability of metallic materials, they reduce the products’ production rate and limit the thermomechanical processing of metallic materials, within a certain degree. Moreover, the quality of thermomechanical work-piece is highly dependent on the forming process of the metallic materials, for achieving the desired shapes, without any flow instability, fracturing, and cracking. Titanium alloys are regarded as hard deformed materials and are easy to be cracked and fractured during hot processing, due to its high processing parameter sensitivity and relatively narrow processing windows. Near beta titanium alloys are even more sensitive to the processing variables, compared to other titanium alloys. Thus, the cracking behavior and mechanisms of titanium alloys, during hot processing need to be investigated and understood to limit and reduce the cracking phenomenon in the thermomechanical processing of titanium alloys and fabricate the products, with a satisfied shape and properties.

Some research works have been done to investigate the cracking behavior and establish cracking criteria or models for titanium alloys. Zhu et al. [20,21] investigated the cracking behavior and established the cracking criteria for Ti-25V-15Cr alloy, and also a reliable prediction model that inter-relates the processing parameters, and the ductile fracture was established via a high-speed photography technique. The cracking behavior of the alloy exhibited a high sensitivity to the processing parameters, it changed from a brittle to a ductile fracture, with an increase in the processing temperature, and no cracks were found at the deformation strain rate of 0.01 s^−1^. Semiatin et al. [22], reported a free-surface cracking, when hot forging the α-annealed Ti-6Al-4V alloy, at just above its β transus temperature; they believed that the cracking was generated by the cavity initiation near the material’s surface. Katani et al. [23] studied the failure mechanism of the mill-annealed Ti-6Al-4V alloy, using the simulation method developed on the basis of the finite element models, and found that the ductile cracking of the material was caused by the micro-void in the α phase of the α/β interface.

However, few research studies have been conducted on the cracking and fracturing behavior of PM metallic materials and no literature refers to the cracking behavior and mechanism of the PM titanium alloys, during hot processing. In this work, the cracking behavior and mechanism of the PM Ti-5553 alloy, prepared via a fast consolidation approach, were investigated at various hot processing conditions, by comparing the results obtained from the ingot metallurgy Ti-5553 alloy. The aim was to provide theoretical support for the titanium industry, to implement a precise hot processing of the PM Ti-5553 alloy.

## 2. Materials and Methods

The raw materials for synthesizing a PM Ti-5Al-5Mo-5V-3Cr (wt.%) alloy in this study were hydride–dehydrided (HDH) titanium powder (Ti > 99.6%, −200 mesh), Al powder (Al > 99.5%, −200 mesh), and master alloy powders (Al35-V65, Al15-Mo85 and Al30-Cr70 in wt.%, 75 μm, −250 mesh) supplied by the Dalian Rongde company (PRC) (Dalian, China). A V-shape blender (Rongde Ltd.) was used to mix the raw powders with the target nominal composition, at a speed of 60 rpm, for 1.5 h. After that, every 500 g powder mixture was warm-pressed into a cylinder green powder compact, with a diameter of 56 mm and a relative density of 83%, at about 250 °C, under a uniaxial pressure of about 400 MPa in air; then the powder compact was consolidated by the modified hot-pressing process. Before the hot-pressing, graphite cream was painted on the in-wall of the mold, to serve as a lubricant. The green compact was heated up, rapidly, in an induction coil to the target temperature of 1250 °C to 1300 °C, in the protective argon atmosphere, followed by a hot-pressing under uniaxial pressure. Hereinafter, the billet was cooled down to room temperature in the flow argon protection, the detailed processes of this rapid consolidation approach have also been described elsewhere [18,19,24]. The consolidated PM Ti-5553 alloy billet had a diameter of 58 mm and a relative density of 98%. The ingot metallurgy Ti-5553 alloy billet was cut from a 20 kg as-received IM Ti-5Al-5Mo-5V-3Cr (wt.%) ingot manufactured through conventional double vacuum arc remelting (VAR) and casting. The actual chemical compositions of the as-consolidated PM and as-cast IM Ti-5553 alloy, were measured by a chemical titrimetric analysis method (inductively coupled plasma atomic emission spectrometry); the results are found in Table 1. It was clear that the major difference of the chemical composition between the PM and the IM alloy, was the oxygen content—0.08% in the IM alloy and 0.36% in the PM alloy. In addition, the as-cast IM alloy had a slightly higher alloying element content (in Al, Mo, V, and Cr) than those of the as-consolidated PM alloy. The beta transus temperature of the PM alloy and the IM alloy was about 975 °C and 875 °C, respectively, which was determined by heat treatment and metallographic methods (heating and water-quenching the specimens to decide the lowest heating temperature, without any α precipitate in the microstructure).

The cylindrical specimens were first wire-cut (EDM), from, both, the as-consolidated PM and as-cast IM Ti-5553 alloys, and then the specimens were machined to the right dimensions (10 mm in diameter and 15 mm in height), for the hot compression tests. The isothermal compression tests of the PM alloy were carried out on a Gleeble^®^ 3800-GTC thermal physical simulator (Dynamic Systems Inc., Austin, TX, USA), under vacuum, at a temperature range of 600 °C–850 °C, and a strain rate range of 0.1^−1^ s^−1^–10 s^−1^. A thermocouple was welded onto the middle surface of the specimens, to capture the deformation temperature during hot compression, and tantalum foils were used to reduce the friction and improve the deformation homogeneity. Before starting the compression, the specimens were first heated up to the desired temperature, at a rate of 10 °C/s, and then the temperature was held constant for 4 min, to assure thermal equilibrium. The total compression deformation of the specimens was about 70% of height reduction, which corresponded to a true strain of 1.2. After the compression, the specimens were immediately quenched in water to keep the deformed microstructures. For the IM Ti-5553 alloy specimens, the isothermal compression tests were carried at 700 °C, and strain rate of 0.01 s^−1^–1 s^−1^, following a procedure similar to that of the PM samples.

X-ray diffraction (XRD) analysis was used to determine the phase constitutions of the PM and the IM alloys, using a Bruker D8 Advanced X-ray diffractometer with a Cu Kα radiation source (λ = 0.154157 nm) (Bruker Ltd., Billerica, MA, USA). The microstructure examination and cracking morphology observation were conducted on an PMG3 optical microscope (OM) (Olympus Corporation, Tokyo, Japan) and JSM-6460 scanning electron microscope (SEM) (JEOL Ltd., Tokyo, Japan); the OM and SEM samples for the microstructure examination were prepared following the standard procedure—grinding, polishing, and then etching, using Kroll’s reagent (10 mL HF + 20 mL HNO_3_ + 70 mL H_2_O). For the TEM observation, ion milling (for PM alloy) and twin-jet electron-polishing (for IM alloy) were utilized to produce the TEM samples that were examined by a JEM2100 transmission electron microscope (JEOL Ltd., Tokyo, Japan).

## 3. Results and Discussions

### 3.1. Initial Microstructures

**Figure 1:**
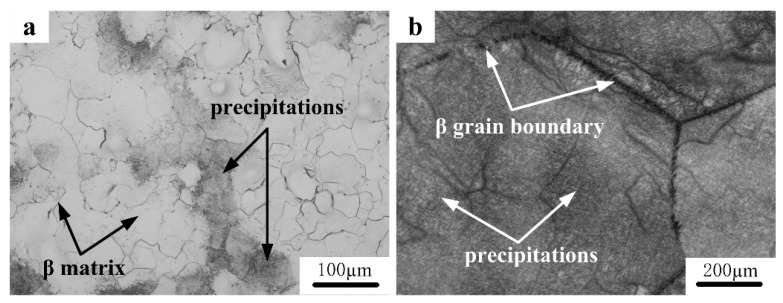
Initial microstructures of the Ti-5553 alloys: (**a**) As-consolidated powder metallurgy (PM) alloy; and (**b**) as-cast ingot metallurgy (IM) alloy.

The initial microstructures of the PM and IM Ti-5553 alloys are shown in Figure 1. Much finer microstructure can be found in the PM alloy (Figure 1a showing an average grain size of 100 μm) than the IM alloy (Figure 1b, showing an average grain size of 1000 μm), and some residual pores can be identified in the microstructure of the PM alloy. A typical β grain matrix can be observed in, both, the PM and the IM alloy, and the IM alloy was composed of a large number of dispersed α phase, which was spread over a β matrix, while only a small amount of the agminated α precipitates could be seen in the PM alloy, mainly distributed along β grain boundaries [19,25].

### 3.2. X-ray Diffraction Patterns

**Figure 2:**
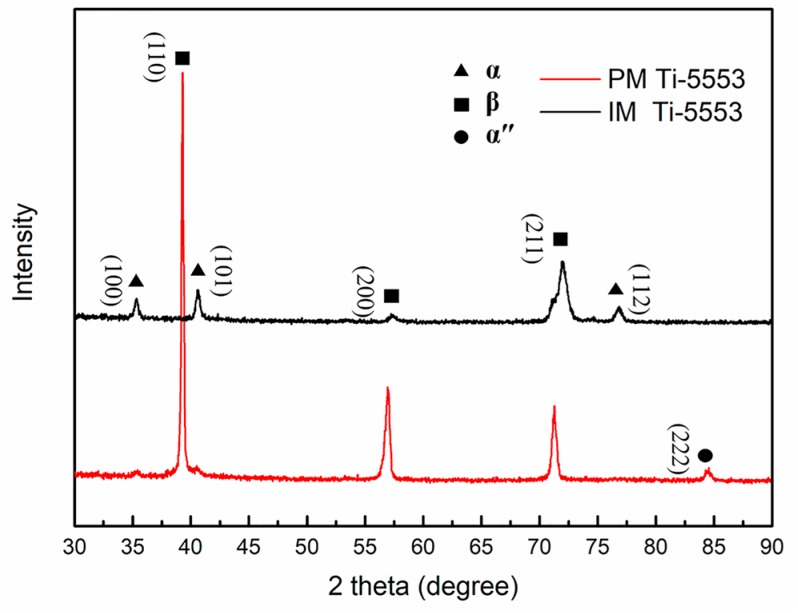
XRD patterns of the as-consolidated PM and the as-cast IM Ti-5553 alloys.

Figure 2 shows the XRD patterns of the PM and the IM Ti-5553 alloys. It is clear that, for the PM alloy, mainly β peaks (very weak α and α″ peaks) appeared in the XRD pattern. Combining the observation from Figure 1a, it could be inferred that the PM alloy was mainly composed of the β phase, and the amount of α precipitates was very small, so it was not clearly reflected in the XRD pattern. As for the IM alloy, both α and β peaks could be clearly identified; the intensity of the β peaks was obviously weaker and the intensity of the α peaks was stronger than the PM alloy. This indicates a larger α phase but smaller β phase had formed in the IM alloy, than in the PM alloy. This could be attributed to the difference in the cooling rates after powder consolidation and ingot casting. For the PM Ti-5553 alloy, the powder compact was about 500 g and the hot-pressing temperature of 1250 °C was higher than the alloy’s β transus temperature of 975 °C, so that the hot-pressed billet could be rapidly cooled down to the room temperature, by flow-argon cooling, after consolidation. The α phase surpassed the precipitate from the β phase, in the cooling process, leading to only a short α phase in the PM alloy. However, the Ti-5553 ingot was about 20 kg and the ingot furnace cooled down to the room temperature, after casting; the cooling rate was very slow and was close to an equilibrium state. Therefore, the α phase had enough time to precipitate from the β phase, in the cooling process, resulting in a large amount of α phases that were formed in the IM alloy.

### 3.3. The Cracking Behavior of the As-Consolidated PM Ti-5553 Alloy

**Figure 3:**
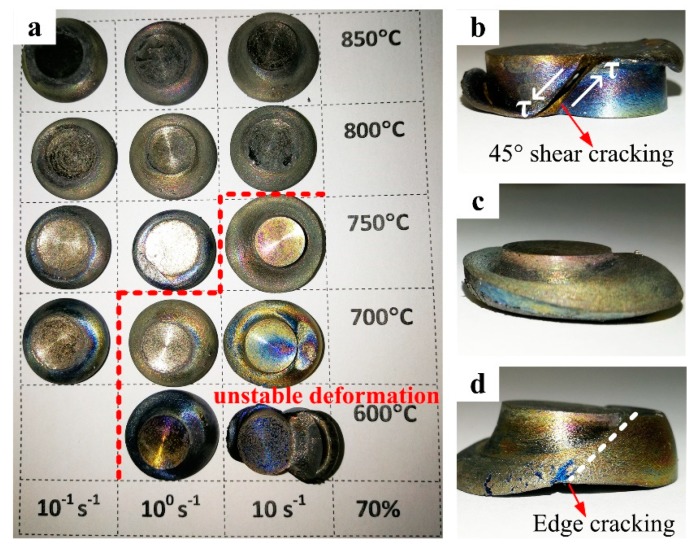
Macroscopical images of the hot-compressed PM Ti-5553 specimens, under various conditions at: (**a**) 600 °C–800 °C/0.1 s^−1^–10 s^−1^; (**b**) 600 °C/10 s^−1^; (**c**) 600 °C/1 s^−1^ and (**d**) 700 °C/10 s^−1^.

The macroscopical views of the PM Ti-5553 alloy specimens, deformed at different hot compression conditions (600 °C–850 °C, 0.1 s^−1^–10 s^−1^), are shown in Figure 3. It was obvious that the cracking behavior just occurred for the specimens compressed at 600 °C/10 s^−1^, with a 45° shear fracture (Figure 3b), and at 700 °C/10 s^−1^, with edge-cracking, accompanied by the 45° shear fracture tendency (Figure 3d). The specimens compressed at other conditions were free of the external cracking. However, for the specimens compressed at the strain rate of 1 s^−1^ at 600 °C and 700 °C, obvious flow instability features could be seen. These suggest that the degrees of cracking were sensitive to both the strain rate and the temperature, the lower the deformation temperature and the higher the deformation rate, the higher was the possibility of cracking for the compressed specimens.

Forty-five degrees shear fracture of the material was formed on the basis of 45° shear bands, which were initiated at the center of the specimen and propagated along the maximum shear stress direction. The crack’s expansion velocity had increased with an increasing strain rate, this explained how the cracking mode was transformed for the compressed specimens, at 600 °C, from edge-cracking (1 s^−1^, Figure 3c) to a 45° shear fracture (10 s^−1^, Figure 3b).

Although no cracks were found at the other conditions, evident unstable, and inhomogeneous deformation features could be observed in the specimens compressed at 600 °C/1 s^−1^, 700 °C/1 s^−1^ and 750 °C/10 s^−1^ (Figure 3a, on the right side of the red dashed line). Furthermore, cracks could be eliminated and the specimen deformation tended to become more homogenous and stable, with increasing plasticity and hot workability, due to the increasing compression temperature and decreasing deformation strain rate. A homogenous and stable deformation could be seen for the specimens compressed at a strain rate of 0.1 s^−1^ at 800 °C and 850 °C.

**Figure 4:**
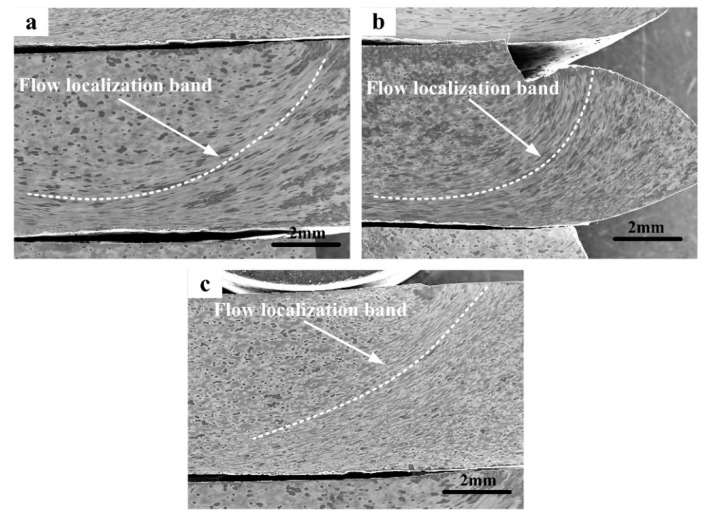
SEM images showing the flow localization situations of the unstable deformed PM Ti-5553 alloy specimens: (**a**) 700 °C/10 s^−1^; (**b**) 600 °C/1 s^−1^ and (**c**) 700 °C/1 s^−1^.

As shown in Figure 4, plastic flow localization bands, with typical unstable and inhomogeneous deformation, could be seen in the specimens compressed at 700 °C /10 s^−1^, 600 °C /1 s^−1^, and 700 °C/1 s^−1^, respectively. The effect of the deformation parameters on the degree of flow localization, was significant, the most serious localized plastic flow could be found in the specimen compressed at 700 °C/10 s^−1^ (Figure 4a), and the localized plastic flow got weaker, when the specimen was compressed at 600 °C/1 s^−1^ (Figure 4b) and even weaker when compressed at 700 °C/1 s^−1^ (Figure 4c). This phenomenon could be attributed to the larger heat generation and greater rise in temperature when the specimens were compressed at a high strain rate and a low temperature, than that at a low strain rate and high temperature [26].

**Figure 5:**
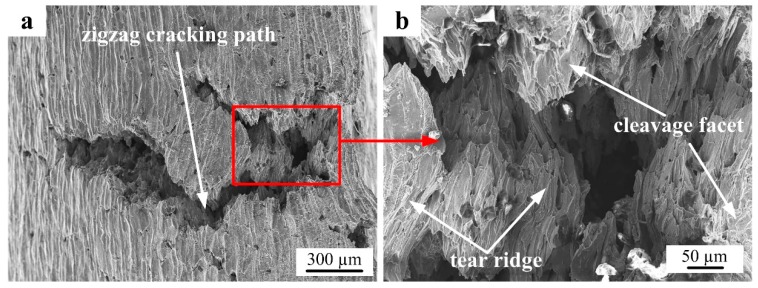
SEM images showing cracking morphology on the compressed PM Ti-5553 alloy specimen at 700 °C/10 s^−1^.

The cracking mechanism of the PM alloy was revealed by investigating the detailed morphology of the cracking area of the specimen compressed at 700 °C/10 s^−1^. As shown in Figure 5a, deep cracks with a zigzag cracking path could be seen, and a more detailed cracking area was observed (as shown in Figure 5b); the appearances of cleavage facets, tear ridges and river like patterns were obvious. These suggest that the brittle cleavage trans-granular cracking mechanism was dominant and the PM Ti-5553 alloy showed little plastic deformation, in the compression condition of 700 °C/10 s^−1^.

### 3.4. The Cracking Behavior of the As-Cast IM Ti-5553 Alloy

**Figure 6:**
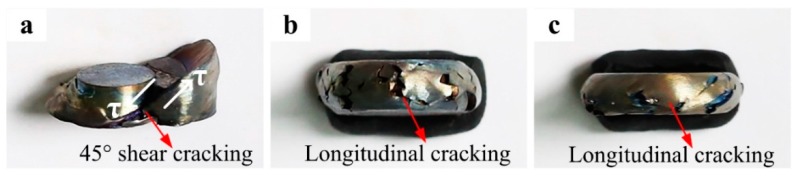
The fracture and cracking modes of the compressed IM Ti-5553 alloy specimens at various conditions: (**a**) 700 °C/10 s^−1^; (**b**) 700 °C/1 s^−1^; and (**c**) 700 °C/0.01 s^−1^.

The fracture and cracking situations of the IM Ti-5553 alloy specimens, hot compressed at 700 °C and various strain rates of 10 s^−1^, 1 s^−1^, and 0.01 s^−1^, are shown in Figure 6. It is clear that the cracking occurred in all compressed specimens, and the degree of fracturing and cracking of IM alloy specimens decreased with a decreasing strain rate. A 45° shear fracture could be seen in the specimen compressed at 10 s^−1^ (Figure 6a), and the specimens exhibited a free-surface longitudinal cracking, when compressed at strain rates of 1 s^−1^ (Figure 6b) and 0.01 s^−1^ (Figure 6c). These results demonstrated that the macroscopical fracturing and cracking mechanism of the IM alloy was highly dependent on the strain rate. Unlike a 45° shear fracture, the free-surface longitudinal cracking was mainly caused by the secondary tensile stresses. These stresses mainly came from the upsetting of the specimen, during the uniaxial hot compression, and they were applied perpendicular to the compression direction [12].

**Figure 7:**
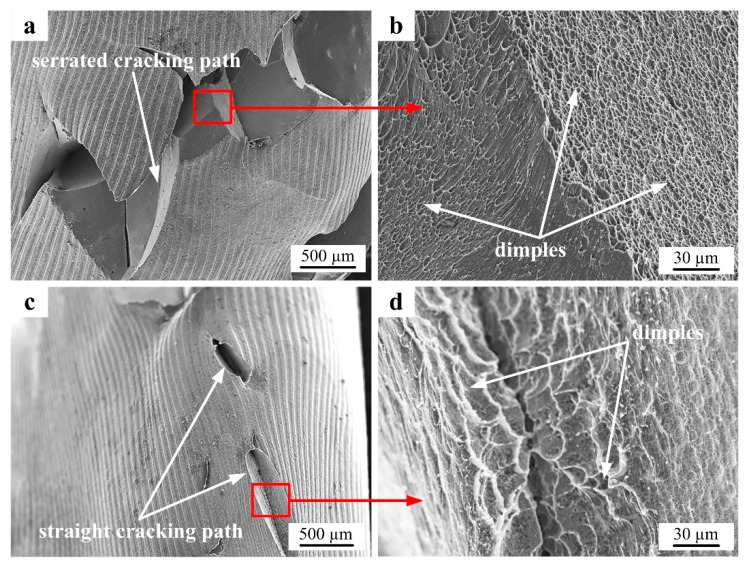
SEM images showing the cracking morphologies of the compressed IM Ti-5553 alloy specimens: (**a**,**b**) 700 °C/1 s^−1^; and (**c**,**d**) 700 °C/0.01 s^−1^.

The detailed morphologies of the cracking areas for the IM Ti-5553 alloy specimens compressed at 700 °C/1 s^−1^ and 700 °C/0.01 s^−1^ are shown in Figure 7. A serrated trans-granular cracking path could be easily seen for the specimen compressed at 700 °C/1 s^−1^ (Figure 7a), but the cracking surface was very smooth and had small and shallow dimples (Figure 7b). For the specimen compressed at 700 °C/0.1 s^−1^, the cracking path was straight, however, the cracking surface was rough and spread with large and deep dimples. This indicated that the IM Ti-5553 alloy had a ductile cracking characteristic. Moreover, it could be noticed that the cracking ductility of the IM alloy at 700 °C, was more prominent when the deformation strain rate was low. This was mainly attributed to the crack expansion velocity which was slow when the deformation strain rate was low, leading to only a slight free-surface longitudinal cracking observed for the specimen compressed at 700 °C/0.1 s^−1^.

### 3.5. Flow Behavior and Microstructural Evolution of the PM and IM Alloys

**Figure 8:**
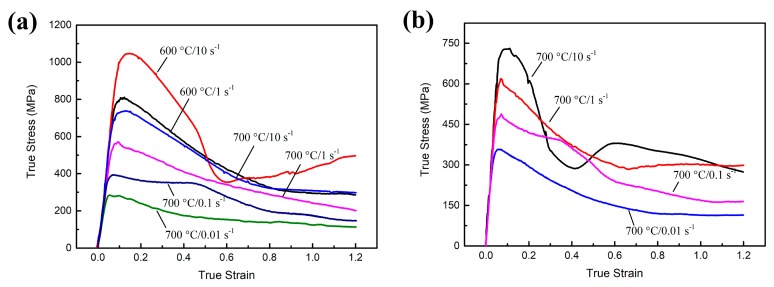
Flow curves of the PM and the IM Ti-5553 alloy at various conditions: (**a**) PM alloy at 600 °C and 700 °C; and (**b**) IM alloy at 700 °C.

The true stress–strain curves of the PM Ti-5553 alloy, compressed at 600 °C and 700 °C, at various strain rates ranging from 0.01 s^−1^ to 10 s^−1^, and the IM Ti-5553 alloy, compressed at 700 °C and strain rates ranging from 0.01 s^−1^ to 10 s^−1^, are shown in Figure 8. As the curves suggest, the flow stress at most compression conditions increased rapidly, at the beginning of the deformation to the peak stress and then decreased gradually to the steady states, with increasing deformation strain. Particularly, for the PM alloy specimens compressed at 600 °C/10 s^−1^ (Figure 8a) and the IM alloy specimen compressed at 700 °C/10 s^−1^ (Figure 8b), the flow stress, after reaching the peak stress, dropped rapidly to a minimum level, and then gradually increased and became stable, with a further increase in the deformation strain.

At the initial stage of deformation, the dislocation density increased rapidly, so that the work hardening is more significant than the dynamic softening, this results in the corresponding true stress–strain curves, that sharply increased with the peak stress. After this, the role of dynamic softening becomes more important with increasing deformation strain, to an extent; then the balance between work hardening and dynamic softening is achieved, then the curves go into a steady stage [21,27]. A severe 45° shear fracture (as illustrated in the Figure 3b and Figure 6a) could have caused the obvious flow instability and subsequent dramatic softening, this accounted for the rapid drops of flow stress, at the conditions of 600 °C/10 s^−1^ and 700 °C/10 s^−1^. Moreover, cracking along 45° and the longitudinal directions of the specimens (as shown in Figure 3 and Figure 6), were the reasons to cause a flow softening, under other deformation conditions, in particular at a low temperature and high strain rate deformation.

Meanwhile, it was clear that the deformation parameters had remarkable effects on the flow behavior of both the PM and the IM alloys. Generally, the flow stress and the degree of flow softening increased with increasing strain rates and declining deformation temperatures. There were several reasons for this: (1) when the deformation happened at a high strain rate, the dislocation generation and multiplication were more facilitated and rapid than that at a relatively low strain rate, this led to a high flow stress at a high strain rate; (2) the mobility and annihilation of dislocation, as well as the thermal diffusion process were significantly promoted, at a high deformation temperature, so that low flow stress could be observed at a high-temperature deformation [8,10,28].

**Figure 9:**
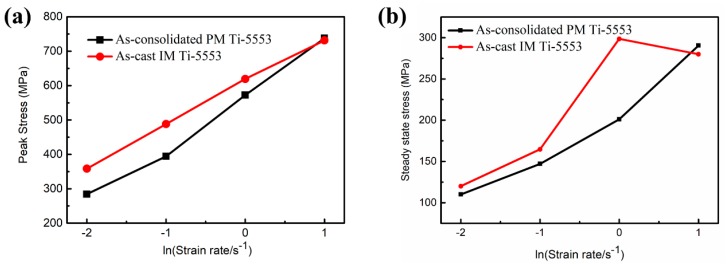
Comparison of the flow stress of the PM and the IM Ti-5553 alloys, at 700 °C, and various strain rates: (**a**) peak stress; and (**b**) steady-state stress (*ε* = 1.2).

In order to investigate the discrepancy of the flow behavior between the PM and the IM alloys, at 700 °C, the peak stress and steady-state stress (*ε* = 1.2) of each condition are plotted in Figure 9. It was clear that, both, the PM and the IM alloys had an almost identical peak stress and steady-state flow stress, at a deformation strain rate of 10 s^−1^. The PM alloy showed edge-cracking (Figure 3d), and 45° shear fracture was observed in the IM alloy (Figure 7a), respectively. However, a peak stress gap could be seen between the PM and the IM alloys, when the deformation strain rate was 1 s^−1^ and it got enlarged at 0.01 s^−1^; the PM alloy also showed a lower peak and a steady-state flow stress than the IM alloy. These results indicated that the PM alloy was much easier to be hot processed at 700 °C than the IM alloy. Meanwhile, the strain rate sensitivity (SRS) parameter, *m*, could be calculated as follows.

(1) $$m={\left(\frac{\dot{\varepsilon }\;\partial \sigma }{\sigma \;\partial \dot{\varepsilon }}\right)}_{T,\varepsilon }={\left(\frac{\partial \;\ln\sigma }{\partial \;\ln\dot{\varepsilon }}\right)}_{T,\varepsilon }$$

**Figure 10:**
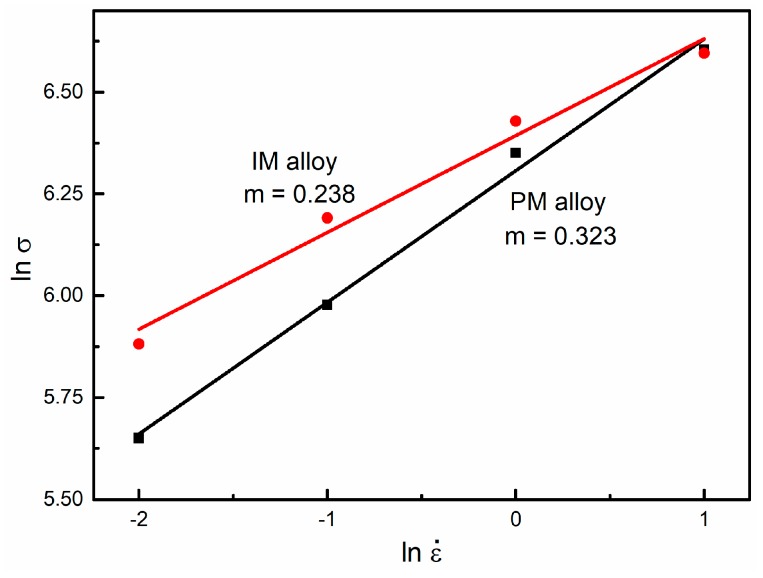
Comparison of the strain rate sensitivity parameter of the PM and the IM Ti-5553 alloys at 700 °C.

The SRS parameter, *m*, demonstrates the hot-workability of the metallic materials and can be obtained from the slope of the linear-fitted (ln *σ* − ln $\dot{\varepsilon }$) graph [29,30], as shown in Figure 10. The PM alloy (*m* = 0.323) displayed a clear higher SRS value than the IM alloy (*m* = 0.238), suggesting a better workability of the PM alloy at 700 °C.

The relatively high flow stress value of the IM alloy at a moderate and low deformation strain rate was attributed to the initial IM alloy, which had a larger grain size, a larger amount of α precipitates, and a shorter β phase than those of the PM alloy. The grains were able to glide along the grain boundaries during the hot deformation, as a result of the viscous flow of grain boundaries [31,32,33], therefore, the finer grain sizes resulted in the lower flow stress of the PM alloy, compared to that of the IM alloy. Furthermore, the crystalline structure of the β phase was body-centered cubic (BCC), in which the deformation took place easily, as the β phase had more active slip systems than the α phase, with a close-packed hexagonal cubic structure (HCP), leading to the dislocations, which could easily glide and climb in the PM Ti-5553 alloy, rather than the IM Ti-5553 alloy [34,35].

**Figure 11:**
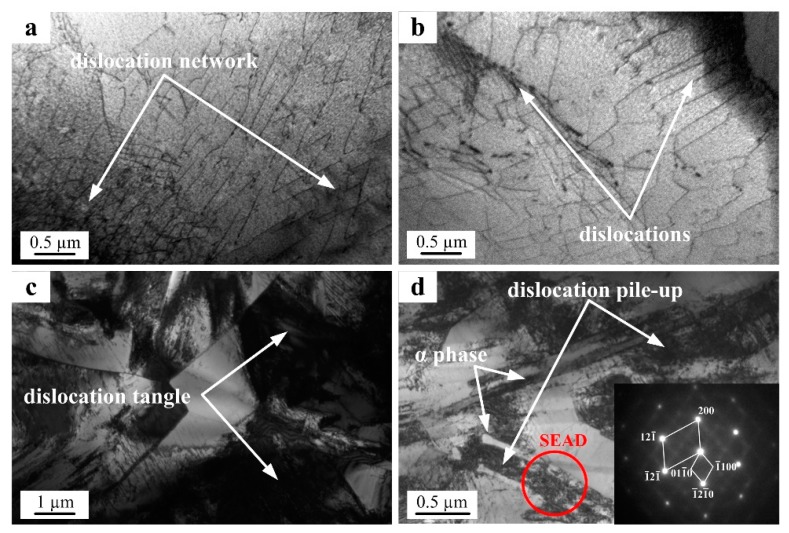
TEM images showing the dislocation configurations and microstructure of the PM and IM Ti-5553 alloys deformed at 700 °C/0.1 s^−1^: (**a**,**b**) PM alloy and (**c**,**d**) IM alloy.

The high-magnification TEM microstructures of the PM and the IM Ti-5553 alloys, after hot-compressing at 700 °C/0.1 s^−1^, are shown in Figure 11. Dislocation networks and mild dislocation pile-up was seen in the PM specimen (Figure 11a,b), whereas, serious dislocation tangle and accumulation could be seen against the grain boundaries in the IM specimen (Figure 11c,d); suggesting that the dislocation was easy to move in the PM alloy and the dislocation movement was significantly prohibited in the IM alloy, at the deformation condition of 700 °C/0.1 s^−1^. Moreover, the dislocation density around the acicular α precipitates (confirmed by the SEAD patterns of the β matrix and α phase precipitates) was high, as can be seen in Figure 10d. This reveals that the dispersed α phase acted as obstacles for the dislocation movement, and thus, the deformation resistance strength of the IM alloy increased [36,37,38]. On the contrary, the higher grain boundary density in the PM alloy acted as dislocation sinks and enabled a higher recovery rate, leading to the elimination of the dislocations, reduction of the flow stress, and improvement of the hot-workability, during the hot deformation.

When the specimens deformed at a high deformation strain rate of 10 s^−1^, the flow stress gap between the IM and the PM alloys disappeared, because the dislocation movement and viscous flow of the grain boundaries did not play dominating roles, in such a very short deformation time, instead, the chemical composition of the alloys became the dominant factor affecting the flow stress, due to the solid solution strengthening effect. As shown in Table 1, the measured actual IM Ti-5553 alloy had higher alloying contents than the PM Ti-5553 alloy, but the PM alloy showed a 0.28 wt.% more oxygen than the IM alloy. The better alloy strengthening effect of the IM alloy than the PM alloy, was counteracted to some extent by the oxygen strengthening in the PM alloy, thus it led to a similar peak flow stress achieved for those two alloys, at a deformation strain rate of 10 s^−1^. However, the effects of the dislocation movement and the grain size became more and more significant with decreasing strain rates; this was reflected by the enlarged gaps between the peak flow stress and steady-state stress of the PM and IM alloys (as shown in Figure 9).

### 3.6. The Comparison of the Cracking Behavior of the As-Consolidated PM Ti-5553 Alloy and the As-Cast IM Ti-5553 Alloy

**Figure 12:**
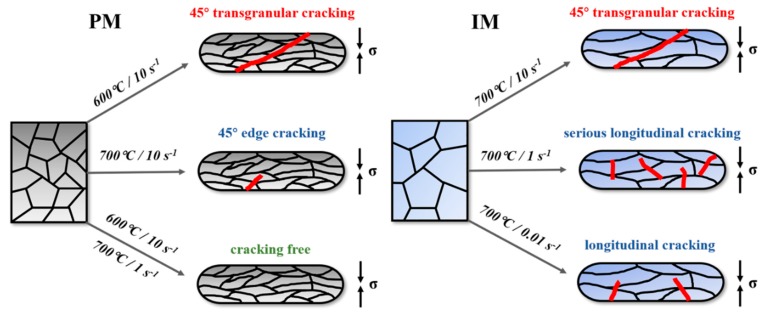
Schematic illustration of the cracking modes of the PM and the IM Ti-5553 alloys.

As shown in Figure 3 and Figure 6, 45° shear cracking occurred in the PM alloy specimen, at 600 °C/10 s^−1^, while the edge cracks could be found in the specimen deformed at 700 °C/10 s^−1^. Moreover, the possibility of cracking and unstable deformation of the PM alloy specimens decreased with increasing deformation temperature and decreasing deformation strain rate. When the strain rate was lower than 10 s^−1^, or the deformation temperature was higher than 700 °C, the crack-free specimens could be produced under any researched conditions. However, cracking still occurred when the IM alloy specimens were compressed at 700 °C, with a 45° shear fracture at 10 s^−1^ and longitudinal cracks at 1 s^−1^ and 0.01 s^−1^. The sketch in Figure 12 illustrates the cracking modes of the PM and IM Ti-5553 alloys, at the conditions discussed above.

Moreover, as discussed in the previous section, the PM Ti-5553 alloy exhibited obvious lower flow stresses than the IM Ti-5553 alloy, under moderate and low strain rate (0.01 s^−1^ to 1 s^−1^) compression conditions. These results indicated that the PM alloy had a better hot-compression workability and higher cracking resistance than the IM alloy. Additionally, the PM Ti-5553 alloy could be hot compressed to a 70% height reduction, without cracking, at a lower temperature (100 °C lower) and a higher stain rate (up to 1 s^−1^) than the IM Ti-5553 alloy.

**Figure 13:**
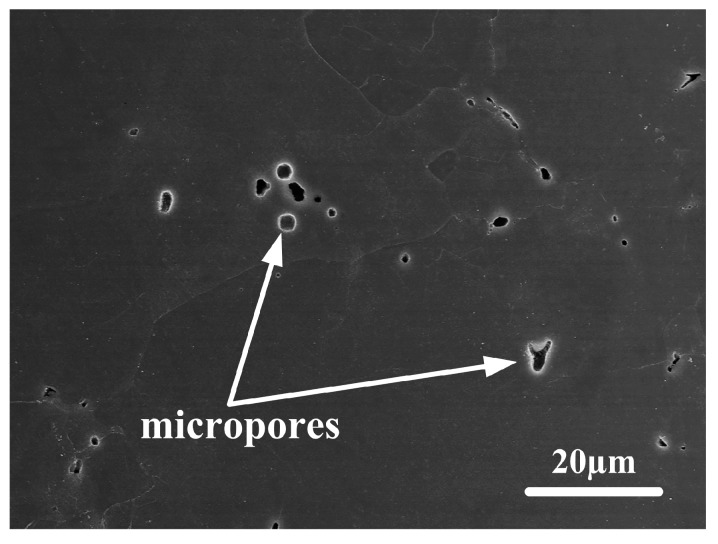
SEM images showing the residual micropores in the microstructure of the PM Ti-5553 alloy.

The PM Ti-5553 alloy exhibited a better thermal–mechanical workability and a higher crack resistance than the IM Ti-5553 alloy, but it showed a lower ductility in the cracking areas. These differences between the PM and IM alloys could be ascribed to the following reasons:

(1) The microstructure of the PM alloy was composed of a primary β phase and very few α precipitates, which provided a better hot deformation ability, and a higher cracking resistance, due to the existence of the BCC crystalline structure (β phase), and an absence of the strengthening effect of the dispersed α precipitates. Meanwhile, the ductility and strength of the alloy were low, due to the nearly single equiaxed β phase, without α precipitate strengthening.
(2) The PM alloy presented more grain boundaries than the IM alloy, which offered an easy path for grain gliding, during hot deformation, leading to a higher cracking resistance for the PM alloy than the IM alloy.
(3) There were still some visible residual micropores, with a size of about 2 μm, in the partial microstructure of the PM alloy (as shown in Figure 13). These micropores could be eliminated and reduced during the hot compression, which improved the cracking resistance and hot-workability for the PM alloy, as the extra energy was absorbed. However, the existence of micropores was detrimental to the ductility, to some extent, as they became the origin of the cracks, in the cracking processes [39].

## 4. Conclusions

Through the above analysis and discussion, the following conclusions could be drawn:

1) The effect of deformation parameters on the cracking behavior of the PM Ti-5553 alloy was significant. Serious fracturing and cracking happened at the conditions of high strain rate (10 s^−1^) and a low deformation temperature (600 °C and 700 °C), while crack-free specimens could be produced when the strain rate was lower than 0.1 s^−1^ and deformation temperature was higher than 750 °C.
2) The PM Ti-5553 alloy had a better hot workability and higher cracking resistance than the IM Ti-5553 alloy, during the hot compression—45° shear cracking occurred for the IM alloy specimens when the deformation condition was at 700 °C/10 s^−1^, however, only an edge-cracking appeared in the PM alloy, at the same condition.
3) The flow stress of the PM Ti-5553 alloy was lower than that of the IM Ti-5553 alloy, at the deformation temperature of 700 °C and moderate or low strain rates, leading to a lower deformation resistance for the PM alloy than the IM alloy.
4) Cleavage brittle fracture features were dominant in the crack surface of the PM Ti-5553 alloy, and ductile dimple cracking characteristics were found in the IM alloy.

## Figures and Tables

**Table 1:** Actual chemical composition of the as-consolidated PM and the as-cast IM Ti-5553 alloy (wt.%).

Alloys	Al	Mo	V	Cr	O	Ti
PM	4.99	4.94	4.93	2.90	0.36	Bal.
IM	5.14	5.02	5.03	3.10	0.08	Bal.
